# Effects of Stretching Speed on Mechanical Rupture of Phospholipid/Cholesterol Bilayers: Molecular Dynamics Simulation

**DOI:** 10.1038/srep15369

**Published:** 2015-10-16

**Authors:** Taiki Shigematsu, Kenichiro Koshiyama, Shigeo Wada

**Affiliations:** 1 Department of Mechanical Science & Bioengineering, Graduate School of Engineering Science, Osaka University, Machikaneyamacho 1-3, Toyonaka, Osaka 560-8531, Japan

## Abstract

Rupture of biological cell membrane under mechanical stresses is critical for cell viability. It is triggered by local rearrangements of membrane molecules. We investigated the effects of stretching speed on mechanical rupture of phospholipid/cholesterol bilayers using unsteady molecular dynamics simulations. We focused on pore formation, the trigger of rupture, in a 40 mol% cholesterol-including bilayer. The unsteady stretching was modeled by proportional and temporal scaling of atom positions at stretching speeds from 0.025 to 30 m/s. The effects of the stretching speed on the critical areal strain, where the pore forms, is composed of two regimes. At low speeds (<1.0 m/s), the critical areal strain is insensitive to speed, whereas it significantly increases at higher speeds. Also, the strain is larger than that of a pure bilayer, regardless of the stretching speeds, which qualitatively agrees with available experimental data. Transient recovery of the cholesterol and phospholipid molecular orientations was evident at lower speeds, suggesting the formation of a stretch-induced interdigitated gel-like phase. However, this recovery was not confirmed at higher speeds or for the pure bilayer. The different responses of the molecular orientations may help explain the two regimes for the effect of stretching speed on pore formation.

Mechanical stresses on biological cell membranes are known to be converted to chemical or electrical signals that regulate various cellular functions, such as cell growth, signal transduction, and transport. In contrast, excessive stresses arising under non-physiological conditions, e.g., in ventricular assisted devices^1^,^2^, during extracorporeal lithotripter^3^,^4^,^5^, or sonoporation treatments^6^,^7^, can induce an irreversible rupture of the cell membrane and subsequent cell death. Understanding the membrane response to mechanical stresses, in particular mechanical rupture, is important for biology as well as in the development of medical devices.

To understand the details of mechanical rupture, many biomechanical experiments have been conducted on biological cell membranes^5^,^8^,^9^,^10^,^11^,^12^ and model membranes consisting of phospholipid bilayers^13^,^14^,^15^,^16^, which are the fundamental structure of biological cell membranes. These elaborate studies have shown that rupture of the membrane occurs when the stress or strain of the membranes exceeds critical values. Under static or at least quasistatic stresses, the rupture tension of biological membranes varies in the range from 1 to 30 mN/m and the rupture areal strain in the range from 0.01 to 0.05 depending on the lipid composition. Needham and Nunn^14^ performed micropipette aspiration experiments on giant bilayer vesicles containing cholesterol molecules at various concentrations and showed that the rupture tension and strain depend strongly on the concentration of cholesterol molecules. In their study, the rupture strains for stearoyloleoylphosphatidylcholine (SOPC) vesicles comprising 38 mol% cholesterol (0.05) was about 1.7 times larger than those of pure SOPC vesicles (0.031). In addition to the lipid composition, the time history of the applied stress or strain affects the rupture stress or strain. Evans and coworkers^13^ also performed micropipette aspiration experiments at various loading rates (0.01–100 mN/m/s) and showed that the rupture tension of vesicles increases with increasing loading rate. The rupture tension for a pure SOPC vesicle, for example, increased 2-fold when the loading rate increased by 3 orders of magnitude^13^. Li and coworkers^10^,^17^ performed impulse-like stretching experiments on red blood cells (RBCs) using a laser-induced cavitation. In their experiments^10^,^17^, the RBCs were rapidly stretched within tens of microseconds and could withstand much higher areal strains of about 0.3, which is about one order of magnitude higher than those in quasistatic stretching experiments. These experiments indicate that membrane rupture is a time-dependent phenomenon and information on the rate at which a membrane is stressed is essential to understanding membrane rupture.

Theoretical and experimental studies have predicted that the rupture of a phospholipid bilayer is initiated by formation of a pore^18^,^19^,^20^,^21^ that penetrates the phospholipid bilayer and is filled with water molecules. According to the model proposed by Lister^19^, the pore is unstable. Depending on the intensity of the applied stresses on the bilayer and the radius of the pore, the pore can spontaneously close or continue to grow indefinitely, leading to rupture of the bilayer. Several experiments have enabled the expanding and closing process of large pores to be directly observed and characterized the dynamics of the pore^22^,^23^. However, because the pore formation itself is an extremely rapid event, triggered by a molecular-scale rearrangement of the membrane structure, it is difficult to capture the details of pore formation in experiments.

Molecular dynamics (MD) simulation of phospholipid bilayers is a great tool that complements experimental observation of such elusive phenomena^24^,^25^,^26^. Many researchers, including our group, have performed MD simulations of the pore formation in the bilayer under various conditions^27^,^28^,^29^,^30^,^31^,^32^. These studies have shown that pore formation is initiated by permeation of water molecules into the hydrophobic interior of the bilayer, which then form a chain-like structure spanning the bilayer. The chain becomes thick and is mostly lined with the hydrophilic head groups of phospholipid molecules, which is a typical (hydrophilic) pore structure. We have performed MD simulations of stretched phospholipid bilayers and investigated the effects of stretching speed^28^ and cholesterol concentration^30^ on pore formation. For pure phospholipid bilayers, the critical areal strain, where a pore is formed, increases with increasing stretching speed, while that under quasistatic stretching also increases with the increasing cholesterol concentration up to 40 mol%. In our simulations, a phospholipid bilayer containing cholesterol at 40 mol% under quasistatic stretching is the toughest composition and forms an interdigitated gel-phase-like structure, in which phospholipid and cholesterol molecules in one leaflet of the bilayer penetrate into the opposite leaflet and become ordered, under stretching. We have proposed a hypothesis to explain the toughness of the phospholipid/cholesterol bilayer in which stretch-induced interdigitation retards pore formation^30^. Although stretch-induced interdigitation has been also observed in some coarse-grained simulations of pure bilayers under tension^33^,^34^, the details of that system are still unknown.

Mammalian cell membranes are usually rich in cholesterol molecules within the phospholipid bilayer^35^. The inclusion of cholesterol in the phospholipid bilayer alters various bilayer characteristics, e.g., lipid fluidity^36^, passive permeability of bilayer^37^, lipid lateral organization^38^, and mechanical stiffness^14^, which are considered to be affected by the ordering of phospholipid molecules in the presence of cholesterol^39^,^40^,^41^,^42^,^43^,^44^. Thus, consideration of the effects of cholesterol at a molecular level is essential to understanding pore formation and subsequent bilayer rupture. Additionally, whereas it has been shown that the stretching speed markedly affects pore formation in a pure phospholipid bilayer, the mechanisms behind the stretching speed effects seen in cholesterol-containing bilayer are still unknown at the molecular level. In particular, the dependence of the stretch-induced interdigitation on the stretching speed must be clarified to understand pore formation under unsteady stretching conditions.

In this paper, we describe a series of molecular dynamics simulations of a dipalmitoylphosphatidylcholine (DPPC) bilayer comprising cholesterol molecules at 40 mol% and a pure DPPC bilayer for comparison under stretching at various stretching speeds. We investigated the effects of stretching speed on pore formation in the DPPC/cholesterol bilayer by analyzing the pore formation patterns, molecular orientations under stretching, critical areal strain where the pore is formed, bilayer thickness, and phase transition to interdigitated gel-like phase under stretching.

## Methods

### Bilayer systems

A planar DPPC/cholesterol bilayer system with a cholesterol concentration of 40 mol% and a pure DPPC bilayer system for comparison were employed. For fair comparison of the stretching speed and to minimize the effects of system size differences between the two systems on the pore formation^32^, we carefully chose the total number of DPPC and cholesterol molecules in the DPPC/cholesterol bilayer to minimize the difference in bilayer areas between the pure DPPC and DPPC/cholesterol bilayers at equilibrium states at 323 K and 1 bar. In this study, the pure DPPC and DPPC/cholesterol bilayers were respectively composed of 128 DPPC and 16,483 water molecules, and 128 DPPC, 86 cholesterol, and 13,842 water molecules, in a square cylinder simulation box with periodic boundary conditions. Here, we set the number of water molecules to prevent the interactions between periodic images of bilayer in the *z*-direction during stretching^28^. DPPC and cholesterol molecules were represented by the united atom force fields for DPPC^45^, and cholesterol^46^ and water molecules by a simple point charge model^47^. For equilibration, constant temperature (*T*) and pressure (*P*) MD simulations were performed for more than 100 ns at 323 K and 1 bar. The areas of the pure DPPC and DPPC/cholesterol bilayers in the equilibrium states were 41.98 and 41.84 nm^2^, respectively, which were essentially as expected. Several structural properties of the pure DPPC and DPPC/cholesterol bilayers in equilibrium states were in agreement with those obtained experimentally^48^,^49^,^50^,^51^ and in other simulations^52^. The details of the construction of the systems, MD simulation parameters for the equilibrium simulation, and the structural properties of the bilayers in equilibrium states are summarized in the supplementary information file. All MD calculations reported here were performed using GROMACS molecular dynamics simulation codes^53^,^54^. All snapshots were rendered using Visual Molecular Dynamics^55^.

### Unsteady stretching simulation

We used the following method^28^ for unsteady stretching (US) of the bilayer, involving proportional and temporal scaling of both the atom positions and system box lengths, implemented in GROMACS codes as the deform option. Briefly, the box lengths *l*_*i*_ and coordinates of all atoms *r* were proportionally scaled per time step *Δt* from *l*_*i*_ to *μl*_*i*_ and *r* to *μr* with


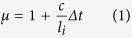


(*i* = *x, y, z*), where *c* is the stretching speed, *c* set to eight values in the range from 0.025 to 30 m/s. Although the stretching speeds here were specified at much higher values than those used during micropipette experiments^13^, we chose these values because of computational limitations. For example, it took about 3,800 CPU hours on a Linux cluster with 2.21 GHz Opteron processors to complete a simulation for a DPPC/cholesterol bilayer with *c* = 0.05 m/s. We used the same value of *μ* in the *x* and *y* directions to express the equibiaxial stretching. However, to maintain the pressure in the *z* direction at a constant value, we applied Berendsen’s scaling factor^56^,


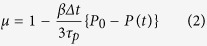


where *τ*_*p*_ = 0.5 ps, *β* = 4.5 × 10^−5^ bar^−1^, and *P*_0_ = 1 bar for scaling in the *z* direction.

During stretching, each temperature for DPPC, cholesterol, and water was individually kept constant at 323 K using the velocity rescaling method^57^ with a 0.2-ps coupling constant. The simulation parameters of the US simulation were essentially the same as those for the equilibrium simulations (see supplementary information file) except for the update frequency of the neighbor list (every 10 steps for *c* < 1.0 m/s and every step for c ≥ 1.0 m/s). To validate the parameters in the US simulations, we performed the same simulations using different parameters: calculation precision (single or double precision), coupling constant *τ*_*p*_ for Berendsen’s scaling factor (0.3–1.0 ps), or number of water molecules (13,842 or 26,183 water molecules). The essential pore formation process was independent of the parameters mentioned above. Because of the statistical nature of pore formation^29^, we performed several replicates of the US simulations, starting from different initial configurations to obtain sample averages. The number of the replicates for the stretching speed conditions is summarized in Table 1.

### Quasistatic stretching simulation

For comparison with the US simulations, a series of constant *NP*_*z*_*A*_||_*T* MD (quasistatic stretching, QS) simulations of the bilayers^28^,^30^,^58^ were performed at constant temperature *T* = 323 K, pressure in the *z* direction *P*_*z*_ = 1 bar, and various constant bilayer areas *A*_||_ that were set to satisfy areal strains up to 1.40, as explained below. We extracted the initial configurations from the trajectories of the US simulations with *c* = 0.05 m/s and performed the simulations using these systems for at least 100 ns so that the systems were equilibrated. We performed the simulations starting from three initial configurations, around the critical areal strains where a pore is formed.

### Analysis

The lengths of the simulation box became *l*_*x*_, *l*_*y*_, *l*_*z*_, and the bilayer thickness *l*_*t*_ upon stretching. The *l*_*t*_ was defined as the distance between phosphorous atoms of DPPC molecules in the upper and lower layers. We defined the bilayer area in the system *A*_||_ as *A*_||_ = *l*_*x*_ × *l*_*y*_ = *l*_*x*_^2^ for *l*_*x*_ = *l*_*y*_ in equibiaxial stretching. We also defined the areal strain of the bilayer *ε*_*A*_ as *ε*_*A*_ = (*l*_*x*_/*l*_*x*0_)^2^ − 1, where *l*_*x*0_ is the average value of *l*_*x*_ during the latter 50 ns of the equilibrium simulation. Ordering in the hydrophobic tails of lipid molecules in the US simulations was evaluated by the averaged instantaneous order parameter 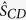
59,


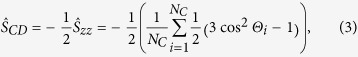


where *Θ*_*i*_ is the angle between the axis of the *i*th molecular axis and bilayer normal (the *z* axis) and *N*_*c*_ is the number of carbons in the lipid chains. In this study, we used the number of carbons in *sn*-2 chains (*N*_*c*_ = 14). For the QS simulation, 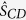 was averaged over time. Furthermore, to analyze the orientations of cholesterol molecules during both the stretching simulations, we defined the cholesterol tilt angle *θ*_*c*_ as the instantaneous angle between the vector linking the C5 and C21 carbon atoms in the cholesterol ring structure and the bilayer normal (the *z* axis). The C5 and C21 atoms are hydrocarbon atoms that both belong to the steroid ring of the cholesterol molecules and are bonded to the oxygen atom of the hydroxyl group and the hydrocarbon chain, respectively.

In our previous QS simulation^30^, the stretch-induced transition to the interdigitated gel-phase-like structure of the DPPC bilayer including cholesterol at 40 mol% is likely to be completed around *ε*_*A*_ = 1.00 through maximization of 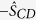. However, such a transition is not observed in pure DPPC bilayers. We assumed that the DPPC/cholesterol bilayer under *ε*_*A*_ = 1.00 forms a standard interdigitated gel-like bilayer, and that the pure DPPC bilayer does not form an interdigitated gel-like bilayer structure under stretching, at least compared with the DPPC/cholesterol bilayer. Based on these assumptions, we defined an evaluation index for the transition to the interdigitated gel-like phase *R*_*Li*_ as 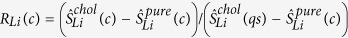. 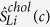 and 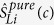 are the order parameter 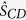 of, respectively, the DPPC/cholesterol and pure DPPC bilayers, where the bilayer thicknesses becomes congruent with that for the DPPC/cholesterol bilayer under *ε*_*A*_ = 1.00. *R*_*Li*_ will take on a value close to 1 when the bilayer forms an interdigitated gel-phase-like structure and will be around 0 when the bilayer dose not, as is the case for the pure DPPC bilayer. Note that the overlap length of the lipid tails that is a measure of the lipid interdigitation shows the similar tendency as *R*_*Li*_ under stretching simulations (see supplementary Fig. S2). Therefore, we use *R*_*Li*_ as an evaluation index for the transition to the interdigitated gel-like phase.

The first step in the pore formation process is the creation of a chain of water molecules penetrating the bilayer^24^,^31^. We defined the areal strain, where the water chain is formed, as the critical areal strain *ε*_*c*_ in the US simulations. Because the applied areal strains were discretized in the QS simulations, we analyzed not only the minimum value of areal strain with a pore, but also the maximum value without a pore in the QS simulation, and the actual *ε*_*c*_ in the QS simulation was expected to be within this range.

Data obtained in unsteady MD simulations, e.g., the critical areal strain, are quite-limited because of the limitation of currently available computational power. To obtain quantitative implications from the limited data, standard statistical analyses were used. The critical areal strains obtained in the US simulations were influenced by two factors: the system composition and the stretching speed. To test these effects, a two-way analysis of variance (ANOVA) on the critical areal strain was performed. When the interaction effect was significant, the tests of the simple main effect and multiple comparison (Ryan’s method) were performed as post-hoc procedures. The differences were considered to be statistically significant at *p* < 0.05.

## Results

### Pore formation process

**Figure 1:**
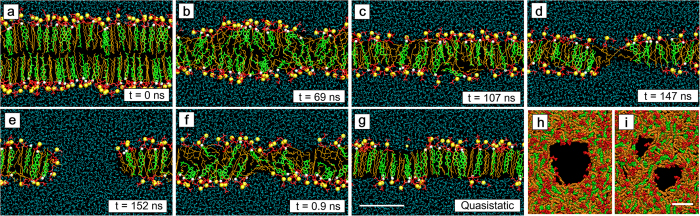
Representative snapshots of DPPC/cholesterol bilayers before stretching (**a**), under unsteady stretching with *c* = 0.025 (**b–e**) and 3.00 m/s (**f,h,i**), and QS simulations (**g**). Panels (**a**–**g**) are side cross-sectional views and (**h**,**i**) are top views. The areal strains of the bilayers are 0.00 (**a**), 0.60 (**b**), 1.00 (**c**,**f**,**g**), 1.44 (**d**), 1.51 (**e**), and 2.40 (**h**,**i**). The DPPC head groups are shown in red, the DPPC tails in orange, the cholesterol molecules in green, the water molecules in blue, the phosphorous atoms in the DPPC molecules as yellow spheres, and the hydrogen atoms in the cholesterol molecules as white spheres. The water molecules in panels (**h**,**i**) are not shown, for clarity. The white bars in panels (**g**,**i**) correspond to 3 nm.

Representative snapshots of the stretched DPPC/cholesterol bilayers in the US and QS simulations are shown in Fig 1. In all stretching simulations, the thickness of the bilayer decreases with stretching (Fig. 1a–c) and, when *ε*_*A*_ exceeds a certain value, which corresponds to *ε*_*c*_, water molecules penetrate into the central part of the bilayer (Fig. 1d). The number of water molecules inside the bilayer quickly increases and a water-filled pore penetrating the bilayer is formed (Fig. 1e). From a visual inspection of snapshots of the DPPC/cholesterol bilayers under stretching, we found three remarkable characteristics of the pore formation process. (i) Just before pore formation, the molecular orientations, e.g., the orientation direction of cholesterol molecules or the configurations of DPPC tails, are more ordered in lower-speed stretching simulations (Fig. 1c,f,g). (ii) The pore formation patterns vary depending on the stretching speed. Two patterns of pore formation in the simulation box were observed in the US simulations; single pore and pores (multi-pore) formations (Fig. 1h,i). In the multi-pore formation, small pores are temporarily formed in small area^28^. The probabilities, where multi-pore formation was observed, are summarized in Table 1. The probability increases with an increase in the stretching speed. (iii) The pore edges of the DPPC/cholesterol bilayer in the US simulations with *c* = 0.025 and 0.05 m/s and the QS simulations are not fully lined with the hydrophilic head groups of lipid molecules (Fig. 1e) and the pore structures are similar to a hydrophobic pore, which is unlike a hydrophilic pore^25^, whose edge is lined in pure phospholipid bilayers. For higher-speed stretching simulation, because the structure of the pore changes rapidly with continuous stretching after pore formation in the system with periodic boundary conditions, the pore edge structure is difficult to examine by visual inspection. In the pure DPPC bilayer, there are several differences from the DPPC/cholesterol bilayer. In particular, the pore structure is a typical hydrophilic pore, the molecular orientations are insensitive to the stretching speed, and multi-pore formation occurs more frequently. The overall tendency in the pore formation process in the pure DPPC bilayer is consistent with the results from our previous US simulations for pure POPC bilayers^28^.

### Molecular orientation

**Figure 2:**
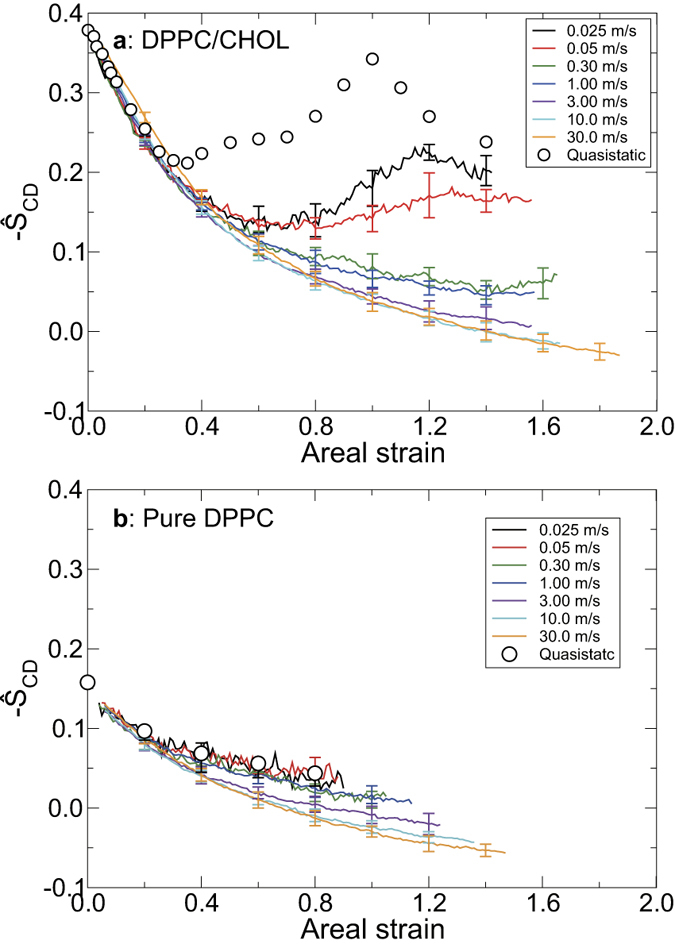
Lipid chain order parameter 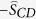 versus areal strain in the DPPC/cholesterol bilayer (a) and the pure DPPC bilayer (b). The error bars represent standard deviation.

From a visual inspection of snapshots of the bilayers, the orientations of DPPC and cholesterol molecules just before pore formation depend upon the stretching speed (Fig. 1c,f,g). As a measure of the molecular orientations of DPPC and cholesterol molecules, the instantaneous order parameter 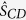 and the cholesterol tilt angle *θ*_*c*_ were evaluated. Figure 2 shows the relationships between 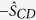 and the areal strain *ε*_*A*_. In the QS simulation for the DPPC/cholesterol bilayer (Fig. 2A), the change in 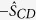 with an increase of *ε*_*A*_ depends strongly on the range of *ε*_*A*_, as we reported previously^30^. 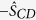 decreases in the range 0.00 ≤ *ε*_*A*_ ≤ 0.30, recovers slightly in the range 0.30 < *ε*_*A*_ < 0.80 and relatively sharply in the range 0.80 ≤ *ε*_*A*_ < 1.00, peaks at *ε*_*A*_ = 1.00, and decreases again over the range 1.00 < *ε*_*A*_ < *ε*_*c*_. In the US simulations with *c* = 0.025 and 0.05 m/s, a similar recovery in 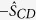 occurs after exceeding *ε*_*A*_ = 0.80, although in the US simulations with c ≥ 0.30 m/s, 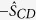 decreases monotonically until pore formation is initiated (*ε*_*A*_ < *ε*_*c*_). For the pure DPPC bilayer (Fig. 2B), 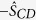 decreases monotonically in all US and QS simulations, and 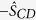 for *c* = 0.025 and 0.05 m/s is similar to that in the QS simulations.

**Figure 3:**
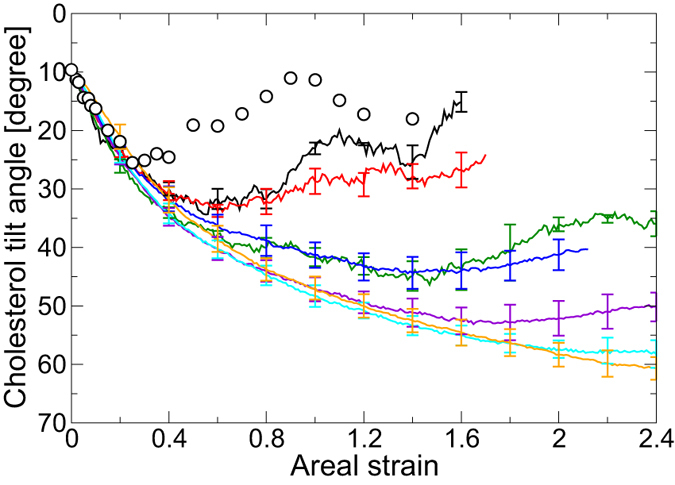
Cholesterol tilt angle versus areal strain. The vertical axis is inverted for convenient comparison with Fig. 2. The error bars represent standard deviation.

Figure 3 shows relationships between the tilt angle of cholesterol molecules, *θ*_*c*_, and the areal strain. (The vertical axis in Fig. 3 is inverted for easy comparison with Fig. 2.) The tendency for *θ*_*c*_ is similar to the inverse for 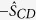. However, *θ*_*c*_ in the QS simulation peaks at *ε*_*A*_ = 0.90, which is slightly smaller than the peak position of 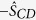.

### Critical areal strain

In the QS simulations for the DPPC/cholesterol bilayer, a pore is formed in all triplicate simulations for *ε*_*A*_ = 1.40, whereas no pore is formed in any of the triplicate simulations for *ε*_*A*_ = 1.20. This indicates that the critical areal strain *ε*_*c*_ in the QS simulation is expected to be in the range 1.20 to 1.40. Similar to the DPPC/cholesterol bilayer, *ε*_*c*_ in the QS simulation for the pure DPPC bilayer is expected to be in the range 0.60 to 0.80.

**Figure 4:**
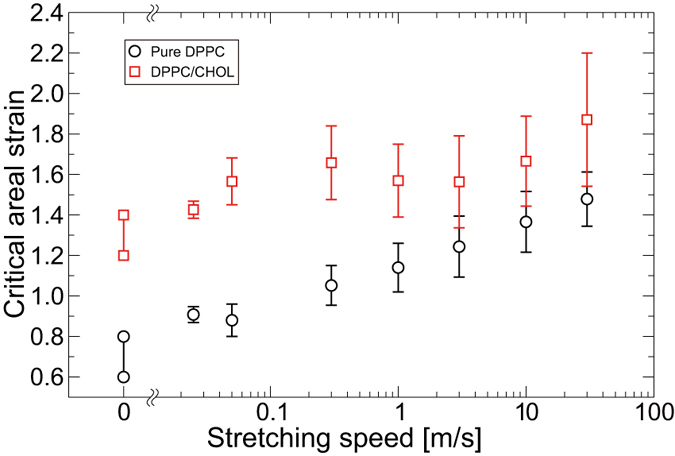
Critical areal strain *ε*_*c*_ as a function of the stretching speed. Minimum areal strain where the pore forms and maximum areal strain where the pore does not form in the QS simulation are shown here at *c* = 0.00 m/s. The error bars represent standard deviation.

Figure 4 shows the relationship between the critical areal strain *ε*_*c*_ and the stretching speed *c* in the US simulations. The maximum areal strains, where a pore is not formed, and the minimum areal strains, where a pore is formed, obtained in the QS simulations are also shown in Fig. 4 for *c* = 0 m/s. For the pure DPPC bilayer, *ε*_*c*_ increases with increasing stretching speed, with a linear curve in semilogarithmic plots. However, for the DPPC/cholesterol bilayer, *ε*_*c*_ non-monotonically changes with increasing stretching speed and tends to increase slightly. At all stretching speeds, *ε*_*c*_ for the DPPC/cholesterol bilayer is larger than that for the pure DPPC bilayer. The two-way ANOVA test indicates that there is a significant interaction effect between the inclusion of cholesterol and the stretching speed (*p* = 0.041). Tests of simple main effect were performed following the statistical procedure described in the Method section above. The tests show that there are significant effects of cholesterol at all stretching speeds (*p* < 0.003) and significant effects of the stretching speed in both pure DPPC (*p* < 10^−4^) and DPPC/cholesterol bilayers (*p* = 0.001). Finally, the multiple comparison tests show that *ε*_*c*_ in the pure DPPC bilayer tend to significantly increase with increasing stretching speed. The pairs of *ε*_*c*_ groups in which there is a significant difference are summarized in the supplementary information file. In the DPPC/cholesterol bilayer, *ε*_*c*_ at *c *= 30.0 m/s is significantly larger than those at *c* = 0.025, 0.05, 1.0, or 3.0 m/s (*p* = 2.3 × 10^−4^, 1.0 × 10^−6^, 0.0017, and 2.0 × 10^−6^, respectively). These statistical analyses clearly show that the effects of stretching speed depend on the range of the stretching speed in the DPPC/cholesterol bilayer, whereas those in the pure DPPC bilayer are monotonic. Additionally, the inclusion of cholesterol increases *ε*_*c*_, regardless of the stretching speed in the range used here. The increasing trend in *ε*_*c*_ with increasing stretching speed and the inclusion of cholesterol are in qualitative agreement with previous simulations^28^,^30^ and experimental studies^13^,^14^. However, it must be noted that the *ε*_*c*_ value obtained here is about two orders of magnitude larger than that obtained from experiments. This discrepancy will be addressed in the Discussion section.

### Bilayer thickness

**Figure 5:**
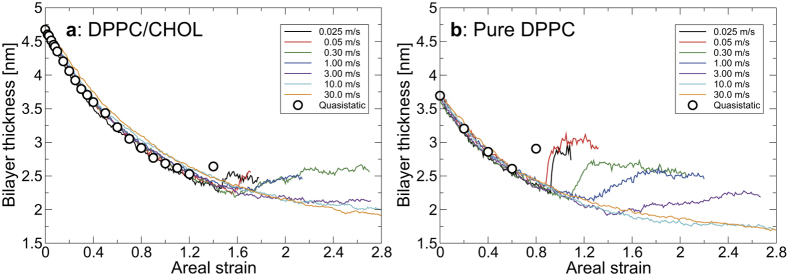
Representative results of bilayer thickness versus areal strain in the DPPC/cholesterol bilayer (**a**) and the pure DPPC bilayer (**b**).

The thickness of the bilayer markedly decreases with stretching (Fig. 1a–d). Figure 5 shows the representative relationship between the bilayer thickness and the areal strain. For both the pure DPPC and DPPC/cholesterol bilayers, *l*_*t*_ decreases with increasing *ε*_*A*_ and there is no difference between the simulations at different stretching speeds before pore formation (*ε*_*A*_ < *ε*_*c*_). After pore formation (*ε*_*A*_ < *ε*_*c*_), *l*_*t*_ for the DPPC/cholesterol bilayer at *c* < 3.0 m/s recovers slightly, but *l*_*t*_ continues to decrease in the other simulations. For the pure DPPC bilayer, the recovery of *l*_*t*_ is observed at *c* < 10.0 m/s and the recovered amount is larger than that for the DPPC/cholesterol bilayer. Under the same areal strain, *l*_*t*_ for the DPPC/cholesterol bilayer without a pore is larger than that for the pure DPPC bilayer.

### Stretch-induced transition to interdigitated gel-like phase

**Figure 6:**
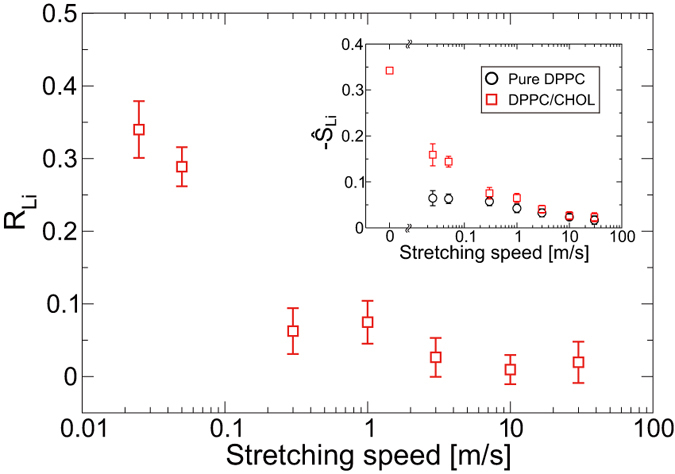
The relationship between the transition index *R*_*Li*_ and the stretching speed. The inset shows the relationship between 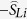 and the stretching speed, which is used to calculate *R*_*Li*_. The error bars represent standard deviation.

The relationships between *R*_*Li*_ and the stretching speed are shown in Fig. 6. With an increase in stretching speed, *R*_*Li*_ in the DPPC/cholesterol bilayer decreases under relatively low-speed stretching (*c* ≤ 0.30 m/s) and, in contrast, does not markedly change under the relatively high-speed stretching (*c* ≥ 0.30 m/s). This trend in *R*_*Li*_ occurs because the difference in the order parameter 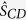 between the DPPC/cholesterol and pure DPPC bilayers, where their thicknesses are the same as that for the interdigitated gel-like bilayer, becomes smaller with an increase in stretching speed (inset in Fig. 6).

## Discussion

The rupture of the bilayer is a time-dependent phenomenon. Pipette aspiration experiments performed by Evans and coworkers^13^ showed that an increase in loading rate induces an increase in rupture tension. They predicted that, at high loading rate, the pore formation during the rupture process becomes the rate-limiting step, which retards the rupture, resulting in an increase in the rupture tension. Assuming a linear relation between tension and strain, our simulation results agree qualitatively with the experimental results. A significant increase in critical areal strain in our simulation is also confirmed (Fig. 4), in spite of the large difference in the range of stretching speed between experiments and simulations. Additionally, the effects of the stretching speed on the critical areal strain in the DPPC/cholesterol bilayer depend upon the range of the stretching speed. The critical areal strain increases at the higher range of stretching speed but does not increase at the lower range. Under lower-speed stretching, the bilayer forms an interdigitated gel-phase-like structure (Fig. 1c,g), followed by the ordering of DPPC and cholesterol molecules (Figs 2a and 3). With an increase in stretching speed, the structural characteristics of the interdigitated gel-like phase becomes ambiguous at the lower range and, in contrast, remain almost constant at the higher range (Fig. 6). Our previous study suggested that stretch-induced interdigitation retards the initiation of the pore formation^30^. Therefore, with an increase in stretching speed at the lower range, pore formation is retarded by its rate-limiting nature and is simultaneously precipitated by the collapse of the interdigitated gel-like bilayer structure, which may be mutually canceled. However, in the higher range, almost no interdigitation occurs and the pore formation is simply retarded by its rate-limiting nature. We deduce that the difference in stretching speed effects on the critical areal strain, which depends on the range of the stretching speed, arises from the dependency on the stretching speed of the stretch-induced ordering with interdigitation.

The critical areal strains of planar lipid bilayers obtained in MD simulations range from ~1 to ~2 (Table 1). The values are about two orders of magnitude larger than those of RBCs and vesicles obtained in micropipette aspiration experiments (0.01–0.05)^14^. Tolpekina and coworkers^32^ showed that the critical areal strain is inversely proportional to the cube root of the reference bilayer area from the free energy model of the bilayer with a pore, considering the finite area in MD simulations. The typical area of the RBCs^60^ and the bilayer vesicle^13^,^14^ in experiments ranges from ~100 to 1000 μm^2^ and that used here is ~42 nm^2^. The range of critical areal strains in our MD simulations becomes the order of 0.001–0.01 by the scaling, which is close to the range in the experiments. However, it should be noted that the model presented by Tolpekina and coworkers does not consider the effects of stretching speeds. It may be required to develop more sophisticated model including the effects of the reference area and the stretching speed^13^ on pore formation, although this is beyond the scope of this paper.

RBCs have a cholesterol-rich membrane, with a cholesterol concentration in the range 40 to 50 mol%^35^, and they have been used as a model of biological cells in various biomechanical experiments^5^,^9^,^10^,^12^. Li and coworkers^10^,^17^ performed impulse-like stretching experiments for RBCs using laser-induced cavitation. They reported that RBCs can withstand higher areal strain (~0.3) than those obtained in micropipette experiments (~0.05). Additionally, leakage of preloaded calcein from RBCs and RBC rupture does not occur immediately after impulsive stretching, but occurs up to tens of seconds after stretching. They suggested that this time lag is caused by the formation of small pores, which are smaller than calcein and are not detectable in optical measurements.

Our previous simulation study showed that multi-pore formation is observed in pure phospholipid bilayers under high-speed stretching^28^. In this study, under higher speed stretching, the same multi-pore formations are observed in cholesterol-including bilayers, whose molecular composition is essentially closer to that of the RBC membrane than that of the pure bilayer. Our results show that, although the inclusion of cholesterol affects several mechanical properties of phospholipid bilayers, multi-pore formation is not predominantly limited by cholesterol and provides the potential for multi-pore formation in biological cell membranes.

## Conclusion

MD simulation of DPPC/cholesterol and pure DPPC bilayers under unsteady stretching at various stretching speeds provides three observations about the critical areal strain, where the pore is formed. These observations are quantitatively confirmed by standard statistical procedures. (i) The effects of stretching speed on the critical areal strain reported in experiments is also observed in our simulation despite the much higher stretching speed compared with those applied in the experiments. (ii) The effects of stretching speed in the DPPC/cholesterol bilayer depend on the range of stretching speed and may arise from the dependence of stretch-induced interdigitation on stretching speed. (iii) The effects of cholesterol are not eliminated even under conditions of extremely rapid stretching.

## Additional Information

**How to cite this article**: Shigematsu, T. *et al.* Effects of Stretching Speed on Mechanical Rupture of Phospholipid/Cholesterol Bilayers: Molecular Dynamics Simulation. *Sci. Rep.* **5**, 15369; doi: 10.1038/srep15369 (2015).

## Supplementary Material

Supplementary Information

## Figures and Tables

**Table 1:** Summary of parameters for the US simulations.

Label	Replicates	Stretching speed [m/s]	*ε*_*c*_ mean ± S.D.	Multi-pore rate
DC0.025	3	0.025	1.43 ± 0.04	0.00
DC0.05	5	0.05	1.57 ± 0.12	0.00
DC0.30	6	0.30	1.66 ± 0.18	0.00
DC1.00	20	1.00	1.57 ± 0.18	0.20
DC3.00	20	3.00	1.56 ± 0.23	0.65
DC10.0	20	10.0	1.67 ± 0.22	0.90
DC30.0	20	30.0	1.87 ± 0.33	1.00
PD0.025	3	0.025	0.91 ± 0.04	0.00
PD0.05	5	0.05	0.88 ± 0.08	0.00
PD0.30	6	0.30	1.05 ± 0.10	0.50
PD1.00	20	1.00	1.14 ± 0.12	0.55
PD3.00	20	3.00	1.24 ± 0.15	0.85
PD10.0	20	10.0	1.37 ± 0.15	0.95
PD30.0	20	30.0	1.48 ± 0.14	1.00
