# Supplementary material for: Effects of Stretching Speed on Mechanical Rupture of Phospholipid/Cholesterol Bilayers: Molecular Dynamics Simulation
Source: Sci Rep. 2015 Oct 16;5:15369. doi: 10.1038/srep15369 (PMC4607938; doi:10.1038/srep15369)
Supplement: Supplementary Information [file srep15369-s1.pdf]

Supplementary information for "Effects of Stretching Speed on Mechanical Rupture of Phospholipid/Cholesterol Bilayers: Molecular Dynamics Simulation"

Taiki Shigematsu, Kenichiro Koshiyama*, and Shigeo Wada

Department of Mechanical Science & Bioengineering, Graduate School of Engineering Science, Osaka University, Machikaneyamacho 1-3, Toyonaka, Osaka 560-8531, Japan

Initial system construction and equilibration

The procedure for the construction of pure DPPC and DPPC/cholesterol bilayer systems, with almost identical planar bilayer areas and square bilayer shapes, is briefly explained in this section. The fundamental procedure is the same as in our previous study[1]. First, two monolayers were constructed by placing DPPC and cholesterol molecules rotated around their long axis on a 8×8 grid. These monolayers contained the same number of cholesterol molecules and the position of each molecule was assigned randomly. The two monolayers were then stacked up to build a bilayer and the entire system was energy minimized. The bilayer was solvated with water molecules, which immersed the polar head group of DPPC and the cholesterol molecules. The solvated system was energy minimized again and equilibrated by *NPT* MD simulation for more than 100 ns. At this point, the pure DPPC bilayer consisted of 128 DPPC and 4,955 water molecules, and the DPPC/cholesterol bilayer of 76

DPPC, 52 cholesterol, and 4,864 water molecules. Additionally, the bilayer areas of the pure DPPC and DPPC/cholesterol bilayer systems at equilibrium state were 41.87 and 25.12 nm^2, respectively. To reduce the difference in bilayer areas, we developed the DPPC/cholesterol bilayer system further. The square DPPC/cholesterol bilayer system was replicated along the direction parallel to the bilayer plain and a rectangular bilayer system was thus formed. The rectangular bilayer system was deformed using the MD simulation with the deform option, implemented in GROMACS codes, to obtain a square bilayer system. Then, three DPPC and two cholesterol molecules per leaflet were randomly removed from the deformed square bilayer system and the system was equilibrated by *NPT* MD simulation for 10 ns. Once again, eight DPPC and six cholesterol molecules per leaflet were removed and the system was equilibrated for 20 ns. The DPPC/cholesterol bilayer consisted of 128 DPPC and 86 cholesterol molecules. Finally, water molecules were added to both the pure DPPC and DPPC/cholesterol bilayer systems. Both the systems were equilibrated using *NPT* MD simulation for more than 100 ns.

Equilibrium simulation parameters

Unless otherwise mentioned, all MD calculations were carried out under the conditions shown below. The leap-frog algorithm was used for numerical solution of the equations of motion and the time step was set to 2.0 fs. The PME method[2] with periodic boundary conditions in all directions was used to calculate the Coulombic interactions with 0.12 nm of the maximum Fourier spacing and fitting

function in the fourth order. A 1.0-nm cutoff was employed for both van del Waals and short-range Coulombic interactions. The neighbor list was updated every 10 steps and the center of mass motion was removed after every step. All bonds were constrained using the linear constraint solver (LINCS)[3] for the DPPC and cholesterol molecules, and the SETTLE algorithm[4] for the water molecules. The temperatures of DPPC, cholesterol, and water were maintained individually at 323 K using the velocity rescaling method[5] with a 0.2 ps coupling constant. The pressure normal and lateral to the bilayer plane were individually maintained at 1 bar using the Berendsen method[6] with a 0.5-ps coupling constant.

Structural parameters of the bilayers

To validate the bilayer structures of the pure DPPC and DPPC/cholesterol bilayers before stretching, we evaluated the typical structural parameters, i.e., area per molecule, bilayer thickness, and the order parameter for the lipid tails. The areas per molecule are 0.656 and 0.391 nm^2 for the pure DPPC and DPPC/cholesterol bilayers, respectively. These are in good agreement with a recent MD simulation study for a DPPC/cholesterol bilayer at 323 K (0.657 and 0.389 nm^2)[7]. However, the area per molecule for the DPPC/cholesterol bilayer is slightly smaller than those obtained in experiments for similar lipid mixtures[8,9], whereas that for the pure DPPC bilayer is in good agreement with those obtained in experiments (summarized by Nagle and Tristram-Nagle[10]). The bilayer thicknesses are 3.70 and 4.68 nm for the pure DPPC and DPPC/cholesterol bilayers, respectively. As with the area per

molecule, the bilayer thicknesses for both bilayers are in good agreement with those obtained in a recent MD simulation study[7] and that for the pure DPPC bilayer is in agreement with experiments for similar lipid bilayers[9]. However, the thickness of the DPPC/cholesterol bilayer is slightly larger than those obtained in experiments with similar lipid mixtures[9]. The averaged order parameter of DPPC tails are 0.16 and 0.38 for the pure DPPC and DPPC/cholesterol bilayers, respectively. These are in good agreement with the recent MD simulation study[7] and in qualitative agreement with experimental measurements[11]. In summary, whereas the structural parameters for the pure DPPC bilayer are in good agreement with those obtained in experiments, those for the DPPC/cholesterol bilayer are slightly different. In particular, the DPPC/cholesterol bilayer in MD simulation tends to be overly condensed, which may be caused by a mismatch in the force field between the DPPC and cholesterol molecules. Meanwhile, the trends in the bilayer structure changes induced by the inclusion of cholesterol are in agreement with both simulations and experimental studies. In this study, we conclude that the bilayer systems used in this study can represent real bilayers, without departing from the accuracy of the current MD simulation for lipid mixtures.

Detailed results of statistical analyses

In this section, the set of critical areal strains obtained in stretching simulations for a given simulation condition is written as $\varepsilon_c(\phi, c)$. ϕ is a bilayer composition parameter, which is 0 for the pure

DPPC bilayer and 1 for the DPPC/cholesterol bilayer, and c is a stretching speed. For example, the set of critical areal strains in the US simulation with c = 0.025 m/s for the DPPC/cholesterol bilayer is written as $\varepsilon_c(1, 0.025)$. In the results of the statistical procedures, i.e., the two-way ANOVA, the test of the simple main effect, and the multiple comparison test, there are significant differences for the critical areal strain in the pure DPPC bilayer between following pairs: $\varepsilon_c(0, 0.025)$ and $\varepsilon_c(0, c \geq 10.0)$, $\varepsilon_c(0, 0.05)$ and $\varepsilon_c(0, c \geq 3.00)$, $\varepsilon_c(0, 0.30)$ and $\varepsilon_c(0, c \geq 10.0)$, $\varepsilon_c(0, 1.00)$ and $\varepsilon_c(0, 30.0)$, and $\varepsilon_c(0, c \geq 3.00)$ and $\varepsilon_c(0, 30.0)$. This implies that the effects of the stretching speed in the range used here on the critical areal strain are monotonic, except for c = 0.025 m/s, and an increase in stretching speed by about two orders of magnitude induces a significant increase in the critical areal strain.

Overlap of DPPC tails between the upper and lower monolayer

In the interdigitated gel-phase-like structure of the DPPC/cholesterol bilayers under lower speed stretching, the DPPC tails penetrate into the opposite monolayer across the mid-plane of the bilayer, resulting in the overlap of the tails between the upper and lower monolayers (Fig. 1c and g). We defined the overlap length D_{ol} as $D_{ol} = z_{lower} - z_{upper}$, where z_{lower} and z_{upper} are the average z-positions of the terminal methyl groups of the DPPC tails in the lower and upper monolayers, respectively. D_{ol} will be positive when the tails are interdigitated and will be negative when the tails do not. Figure S1 shows relationships between the overlap length D_{ol} and the areal strain. The overlap lengths in both the pure

DPPC and DPPC/cholesterol bilayers are about −0.4 nm before stretching and increase with stretching. In the DPPC/cholesterol bilayer, those during the QS simulation and the US simulations with $c = 0.025$ and 0.05 m/s considerably increase and become positive. Especially, that in the QS simulation is about 1.3 nm at $\varepsilon_A = 1.00$. However, those with $c > 0.30$ m/s remain negative during the simulations before the pore formation. In the pure DPPC bilayer, those with $c > 0.30$ m/s remain negative and those with $c \leq 0.30$ m/s slightly exceed 0.

Figure S2 shows the relationships between the stretching speed and the overlap length where the bilayer thickness becomes congruent with that for the DPPC/cholesterol bilayer under $\varepsilon_A = 1.00$. The overlap length in the DPPC/cholesterol bilayer considerably decreases under relatively low-speed stretching ($c \leq 0.30$ m/s), becomes negative, and slightly decreases under relatively high-speed stretching ($c > 0.30$ m/s). This trend in the overlap length in the DPPC/cholesterol bilayer is very similar to the trend in R_{Li} (Fig. 6). In the pure DPPC bilayer, the overlap length in the all stretching simulations remains negative.

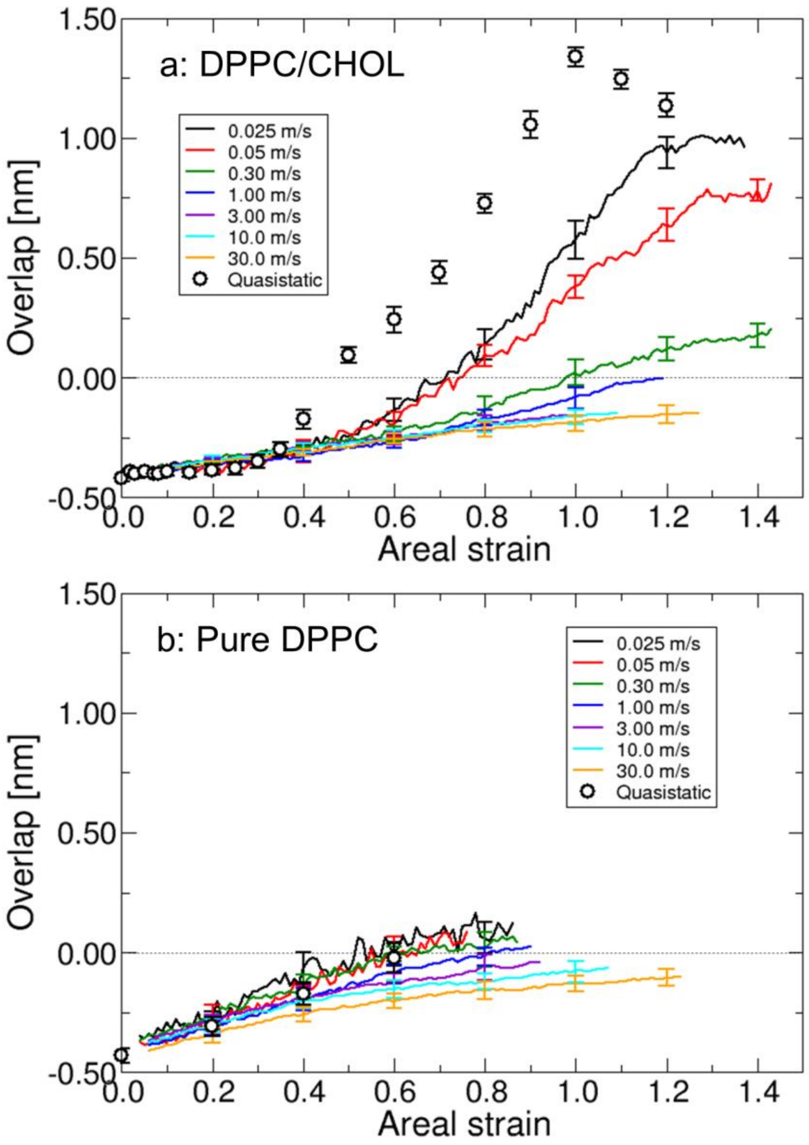

FIGURE S1 The relationship between the overlap length and the areal strain ε_A in the DPPC/cholesterol bilayer (a) and the pure DPPC bilayer (b). The overlap length will be positive when the DPPC tails are interdigitated and will be negative when the tails are not. The error bars represent standard deviation.

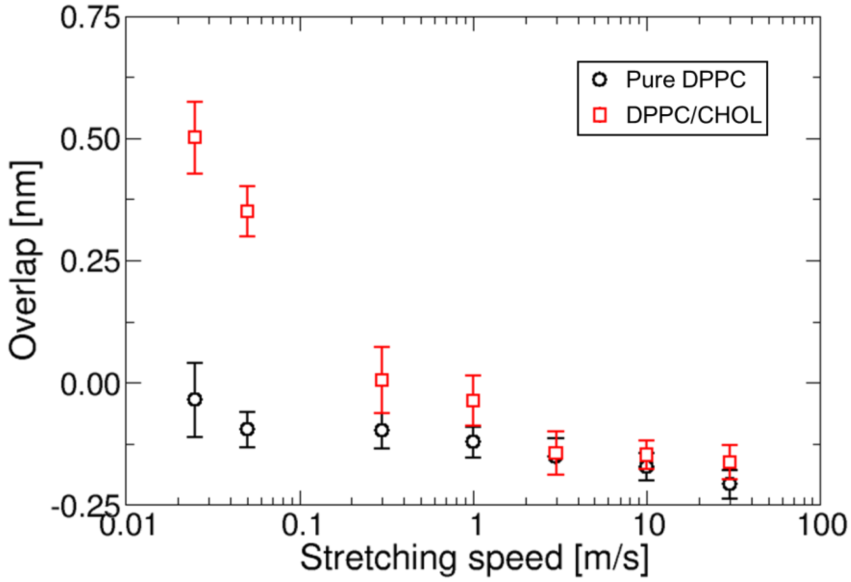

FIGURE S2 The relationships between the stretching speed and the overlap length where the bilayer thickness becomes congruent with that for the DPPC/cholesterol bilayer under ε_A = 1.00. The error bars represent standard deviation.

References

1. Shigematsu, T., Koshiyama, K. & Wada, S. Molecular dynamics simulations of pore formation in stretched phospholipid/cholesterol bilayers. *Chem. Phys. Lipids* **183,** 43–49 (2014).

2. Essmann, U. *et al.* A smooth particle mesh Ewald method. *J. Chem. Phys.* **103,** 8577–8593 (1995).

3. Hess, B., Bekker, H., Berendsen, H. J. C. & Fraaije, J. G. E. M. LINCS: A linear constraint solver for molecular simulations. *J. Comput. Chem.* **18,** 1463–1472 (1997).

4. Miyamoto, S. & Kollman, P. a. Settle: An analytical version of the SHAKE and RATTLE algorithm for rigid water models. *J. Comput. Chem.* **13,** 952–962 (1992).

5. Bussi, G., Donadio, D. & Parrinello, M. Canonical sampling through velocity rescaling. *J. Chem. Phys.* **126,** 014101 (2007).

6. Berendsen, H. J. C., Postma, J. P. M., van Gunsteren, W. F., DiNola, A. & Haak, J. R. Molecular dynamics with coupling to an external bath. *J. Chem. Phys.* **81,** 3684–3690 (1984).

7. Saito, H. & Shinoda, W. Cholesterol effect on water permeability through DPPC and PSM lipid bilayers: a molecular dynamics study. *J. Phys. Chem. B* **115,** 15241–15250 (2011).

8. McConnell, H. & Radhakrishnan, A. Condensed complexes of cholesterol and phospholipids. *Biochim. Biophys. Acta* **1610,** 159–173 (2003).

9. Hung, W. C., Lee, M. T., Chen, F. Y. & Huang, H. W. The condensing effect of cholesterol in lipid bilayers. *Biophys. J.* **92,** 3960–3967 (2007).

10. Nagle, J. F. & Tristram-Nagle, S. Structure of lipid bilayers. *Biochim. Biophys. Acta - Rev. Biomembr.* **1469,** 159–195 (2000).

11. Mills, T. & Toombes, G. Order parameters and areas in fluid-phase oriented lipid membranes using wide angle X-ray scattering. *Biophys. J.* **95,** 669–681 (2008).
